# Supplementary material for: Erector Spinae Plane Blocks for the Early Analgesia of Rib Fractures in Trauma (ESPEAR): protocol for a multicentre pilot randomised controlled trial with feasibility and embedded qualitative assessment
Source: BMJ Open. 2022 Sep 21;12(9):e062935. doi: 10.1136/bmjopen-2022-062935 (PMC9494559; doi:10.1136/bmjopen-2022-062935)
Supplement: Author's manuscript [file bmjopen-2022-062935.draft_revisions.pdf]

BMJ Open is committed to open peer review. As part of this commitment we make the peer review history of every article we publish publicly available.

When an article is published we post the peer reviewers' comments and the authors' responses online. We also post the versions of the paper that were used during peer review. These are the versions that the peer review comments apply to.

The versions of the paper that follow are the versions that were submitted during the peer review process. They are not the versions of record or the final published versions. They should not be cited or distributed as the published version of this manuscript.

BMJ Open is an open access journal and the full, final, typeset and author-corrected version of record of the manuscript is available on our site with no access controls, subscription charges or pay-per-view fees (http://bmjopen.bmj.com).

If you have any questions on BMJ Open's open peer review process please email info.bmjopen@bmj.com

BMJ Open

Erector Spinae Plane blocks for the Early Analgesia of Rib fractures in trauma (ESPEAR): protocol for a multicentre pilot randomised controlled trial with feasibility and embedded qualitative assessment

Journal:	*BMJ Open*
Manuscript ID	bmjopen-2022-062935
Article Type:	Protocol
Date Submitted by the Author:	15-Mar-2022

Complete List of Authors:	Hewson, David; Nottingham University Hospitals NHS Trust, Department of Anaesthesia; University of Nottingham<br>Nightingale, Jessica; Nottingham University Hospitals NHS Trust, Trauma and Orthopaedics<br>Ogollah, Reuben; University of Nottingham University Park Campus, Nottingham Clinical Trials Unit<br>Ollivere, Benjamin; University of Nottingham, Trauma Outcomes Group<br>Costa, Matthew; Oxford University, Nuffield Department of Orthopaedics, Rheumatology, and Musculoskeletal Sciences<br>Craxford, Simon; University of Nottingham, Trauma and orthopaedics<br>Bates, Peter; Barts Health NHS Trust<br>Bedforth, Nigel; Nottingham University Hospitals NHS Trust, Department of Anaethesia

Keywords:	ANAESTHETICS, TRAUMA MANAGEMENT, PAIN MANAGEMENT, ACCIDENT & EMERGENCY MEDICINE

SCHOLARONE™
Manuscripts

Erector Spinae Plane blocks for the Early Analgesia of Rib fractures in trauma (ESPEAR): protocol for a multicentre pilot randomised controlled trial with feasibility and embedded qualitative assessment

David W Hewson,[1,2] Jessica Nightingale,[3] Reuben Ogollah,[4] Ben Ollivere,[2,5] Matthew L Costa,[6] Simon Craxford,[5] Peter Bates,[7] Nigel M Bedforth.[1,2]

1. Department of Anaesthesia, Queen's Medical Centre, Nottingham University Hospitals NHS Trust, NG7 2UH, UK

2. Anaesthesia and Critical Care, Academic Unit of Injury, Inflammation and Recovery Science, School of Medicine, University of Nottingham, Nottingham NG7 2UH, UK

3. Department of Orthopaedic Surgery, Queen's Medical Centre, Nottingham University Hospitals NHS Trust, NG7 2UH, UK

4. Nottingham Clinical Trials Unit, Applied Health Research Building, University Park, University of Nottingham, NG7 2RD, UK

5. Orthopaedic Trauma Surgery, Academic Unit of Injury, Inflammation and Recovery Science, School of Medicine, University of Nottingham, Nottingham NG7 2UH, UK

6. Nuffield Department of Orthopaedics, Rheumatology and Musculoskeletal Sciences, University of Oxford, OX3 9DU, UK

7. Department of Orthopaedic Trauma, Royal London Hospital, Bart's Health, London, E1 1FR.

Corresponding author Dr David Hewson
david.hewson@nottingham.ac.uk
Department of Anaesthesia
C Floor, East Block
Queen's Medical Centre
Nottingham University Hospitals NHS Trust
Nottingham
NG7 2UH
United Kingdom

Word count 3859 words

For peer review only

ABSTRACT

Introduction

Patients with rib fractures commonly experience significant acute pain and are at risk of hypoxia, retained secretions, respiratory failure and death. Effective analgesia improves these outcomes. There is widespread variation in analgesic treatments given to patients including oral, intravenous and epidural routes of administration. Erector spinae place (ESP) blockade, a novel regional analgesic technique, may be effective, but high quality evidence is lacking.

Methods & analysis

To determine if a definitive trial of ESP blockade in rib fractures is possible, we are conducting a multicentre, randomised controlled pilot study with feasibility and qualitative assessment. Fifty adult patients with rib fractures will be randomised in a 1:1 ratio to ESP blockade with multimodal analgesia or placebo ESP blockade with multimodal analgesia. Participants and outcome assessors will be blinded. The primary feasibility outcomes are recruitment rate, retention rate and trial acceptability assessed by interview.

Ethics & dissemination

The study was approved by the Oxford B Research Ethics Committee on 22 February 2022 (REC reference 22/SC/0005). All participants will provide written consent. Trial results will be reported via peer-review and to grant funders.

Registration details

ISRCTN: 49307616. Protocol version 1.2 dated 07/02/22.

KEYWORDS

Rib fractures, chest trauma, regional anaesthesia, analgesia, erector spinae plane block.

For peer review only

STRENGTHS AND LIMITATIONS

- There is widespread variation in the care of patients following rib fractures. The clinical effectiveness of ESP blocks in this patient group is unclear.
- This is a feasibility study with piloting of candidate clinical outcome measures to determine if a definitive trial is feasible; the present work alone cannot answer whether ESP blocks are an effective analgesic modality for patients with rib fractures.
- A feasibility design is a cost-effective and scientifically rigorous method to inform further clinical trial research on a topic, de-risking later work.
- The study will test if an analgesic placebo arm is an acceptable methodological feature for participants, clinicians and investigators.
- The study used a double-blind design and the effectiveness of blinding will be determined by patient and staff interviews.

For peer review only

INTRODUCTION

The pain from rib fractures is often described by patients as the worst pain they have ever experienced. The major complication of this pain is that patients are unable to cough and breathe deeply, causing atelectasis, retained secretions, hypoxaemia, pneumonia and progressive respiratory failure. Deterioration may require mechanical ventilation on an intensive care unit and lead to death.[1,2] This morbidity and mortality is a direct result of severe pain and impaired gas exchange from underlying contused lung parenchyma and altered ventilatory mechanics from the bone injury.[3] The presence of rib fractures in trauma is associated with a significantly increased risk of death, regardless of other injuries, with an odds ratio of 1.4 (95% CI 1.3–1.6) for adults 18-45 years old and 2.5 (95% CI 2.3–2.8) for adults older than 64 years.[4] This injury is therefore particularly devastating for older adults who not only have a higher risk of death but are also likely to sustain rib fractures from less traumatic accidents (due to bone fragility), for example falling from standing height.[5]

A key objective in the multidisciplinary care of people with rib fractures is the assessment and treatment of pain to provide patient comfort and allow normal respiration and cough to minimise the risk of respiratory failure.[3,6] Alongside specialist physiotherapy and daily multidisciplinary review, good pain management is a vital element of early rib fracture care. Despite this, there is no agreement about the optimal pain relief to give patients. The literature on the use of the different analgesic techniques in rib fractures is inconclusive. Although national and international guidance recommends a multimodal approach in preference to opioid medications alone,[7] two meta-analyses concluded that the evidence to recommend any specific treatment modality is insufficient, and that there is no firm evidence for benefit or harm of one analgesic technique over another.[8,9] This leaves clinicians unsure of which analgesic techniques to use. National UK guidance specifies protocolised analgesic regimes as a standard of care for every patient with multiple rib fractures.[10] However the paucity of evidence meant that this guidance could not recommend which analgesic modality (epidural, peripheral nerve blocks or opioid) should be used in which clinical circumstances. Most patients with rib fractures are given a combination of analgesic drugs like paracetamol, non-steroidal anti-inflammatories, opioids and ketamine to help them cope with severe pain; these are the cornerstones of multimodal analgesia in this setting. Medication side effects (including nausea, pruritus, hallucinations, constipation, renal failure and respiratory depression) significantly limit their use. Some patients receive thoracic epidural analgesia (TEA) and some receive other forms of regional

For peer review only

anaesthesia nerve blocks, but the delivery of these interventions by pain-specialist anaesthetists is driven more by local expertise and experience than by high quality evidence.[11]

Regional anaesthesia (including nerve blocks) are clinically useful following rib fractures due to their opioid sparing effect (therefore reducing serious drug related side-effects) and the superior dynamic pain relief they provide. Traditional techniques to block the thoracic nerve supply to the ribs include thoracic epidural analgesia (TEA), paravertebral blockade and intercostal blockade. Systematic review and meta-analysis of these techniques suggested that TEA provides good pain relief, however this benefit does not translate into superior outcomes such as occurrence of pulmonary complications and length of time spent in hospital, intensive care or requiring mechanical ventilation.[12] Unfortunately, TEA has a significant failure rate and is also associated with common and potentially catastrophic complications [13] leading to permanent paralysis, and is therefore contraindicated in approximately one fifth of people with significant injuries. TEA is a complex intervention to perform, practiced by a small and reducing number of anaesthetists nationally and is not available equitably to patients. Even within a single hospital the care delivered varies depending on the time of day and availability of staff to perform such a complex analgesic technique. Only an estimated 9.9-18.4% of patients receive TEA for rib fracture pain.[14]

The erector spinae plane (ESP) block is a regional anaesthetic technique involving the infiltration and infusion of local anaesthetic along fascial planes containing dorsal and ventral rami of thoracic spinal nerves supplying the chest wall.[15] The injection is performed away from the spinal cord (thereby avoiding the complications of TEA). ESP blocks were first described in 2016 [16] and have demonstrated analgesic efficacy for patients on enhanced recovery after surgery protocols (ERAS) following spinal,[17] breast,[18] thoracic [19] and cardiac surgery.[20] In these post-operative acute pain settings, ESP blocks have been shown to reduce patient-reported pain scores and opioid consumption significantly in the early post-operative period compared to multimodal analgesia regimes alone. However, the role of ESP blocks in the management of acute rib fracture pain is currently uncertain.[21] There are no experimental pragmatic multi- centre trials published in this setting, however single-centre cohort data demonstrates ESP blocks provide effective pain relief and improve respiratory function when added to multimodal analgesia in patients with rib fractures.[15,22] Higher quality clinical evidence is urgently needed to guide clinicians on whether the ESP block is a suitable addition to current multimodal analgesia in patients with rib fractures. A definitive trial on this

For peer review only

topic would promote evidence-based practice in rib fracture management and reduce unnecessary variation in clinical practice across UK trauma centres. However there is currently not enough evidence on the effectiveness and acceptability of ESP blocks for rib fractures to undertake a definitive randomised controlled trial (RCT).

The aim of this study is therefore to determine if it is feasible to undertake a definitive RCT to establish if ESP blocks are a clinically effective early treatment for acute pain in patients hospitalised with rib fractures. Formal hypothesis testing for effectiveness or efficacy is not undertaken in feasibility studies. The aim of this trial is not to assess effectiveness or efficacy but to determine feasibility of progression to a definitive RCT.

For peer review only

METHODS AND ANALYSIS

Objectives

This study has the primary objective of determining whether it is feasible to undertake a definitive RCT to establish if ESP blocks are a clinically effective early treatment for acute pain in patients hospitalised with rib fractures. Our primary objectives are to determine:

- Trial recruitment rate.
- Trial retention rate.
- Barriers and facilitators to recruitment and retention among participants and recruitment site staff (anaesthetists, allied health professionals, surgeons and research staff) with regard to the acceptability of the trial intervention.

Secondary trial objectives are:

- To determine the willingness of anaesthetists to randomise patients to intervention or control and willingness of potential participants to randomisation.
- To identify causes of protocol violation and trial withdrawal.
- To assess the completeness of data arising from the trial.
- To assess the fidelity of the trial intervention in terms of ESP catheter dislodgement, blockage or other technical failure.
- To assess the acceptability of the intervention to participants.
- To describe complications of the intervention.
- To pilot the collection of candidate outcome measures for a future definitive trial.
- To determine preliminary indicators of effectiveness as measured by candidate clinical outcome measures.

Patient and Public involvement

The study question builds on previous qualitative work undertaken to validate a patient-derived recovery scale. The scale was developed following interviews with 50 patients and health professionals, with subsequent validation in a 250-patient study. This work characterised the

For peer review only

experience of pain and breathing difficulties following rib fracture; identifying management of pain as a research priority for this patient population. The outcome scale developed through this study will be used as an outcome measure in this trial, to help capture patient-centered outcomes. Specifically for this study we have facilitated virtual focus groups with patients who have previously sustained rib fractures and were admitted to Nottingham University Hospitals NHS Trust. The groups discussed the following aspects: the question and study design; recruitment and consent; follow-up data collection; acceptability of blinding and preferred outcome measures. There was strong support for this study; with individuals acknowledging that pain management of non-operatively managed injuries was an important but often overlooked area of their care. A new regional anaesthetic technique was perceived to be valuable as a treatment option or adjunct since participants said they would be keen to avoid the side-effects associated with oral and intravenous analgesia. The ESP block was perceived by focus group members as less invasive than an epidural. The inclusion of a sham intervention was discussed and deemed acceptable given the integrity of the research. The proposed outcome measures were reviewed by participants and were felt to be comprehensive. They valued the addition of embedded qualitative work within the study to allow for holistic feedback about study acceptability for patients and staff.

Population and setting

The trial will recruit participants at three NHS Major Trauma Centres (MTC):

- Queen's Medical Centre, Nottingham University Hospitals NHS Trust
- John Radcliffe Hospital, Oxford University Hospitals NHS Foundation Trust
- Royal London Hospital, Barts Health NHS Trust

The target population is patients newly admitted to the MTC with one or more new rib fractures who can receive the trial intervention within 12 hours of admission to hospital. Participants will be recruited via their usual clinical care teams (emergency department, major trauma and/or acute pain services), who will notify study investigators of a potentially eligible participant for screening and recruitment purposes.

Inclusion criteria:

For peer review only

- Age ≥ 18
- New admission to major trauma centre and can receive trial intervention within 12 hours of admission
- Mechanism of injury blunt thoracic trauma
- Radiographic evidence of 1 or more new traumatic rib fractures
- Moderate or severe unilateral acute pain (defined as 11-point numerical rating scale (NRS) pain >4 when patient performing vital capacity breath or effective cough) at time of enrolment. Patients may have bilateral fractures, but pain must be unilateral.

Exclusion Criteria:

- Patient refusal or inability to give informed written consent for any reason
- Thoracic injury requiring emergent operative or interventional radiology management
- Allergy to local anaesthetic
- Infection at site of ESP block
- Actual or estimated total body weight ≤ 50 kg thereby precluding safe dosing of local anaesthetic for ESP block
- Current or recent involvement in other clinical research

Interventions and blinding

Following written consent, participant randomisation will be performed to a 1:1 ratio using a web-based automated computer-generated minimisation algorithm with treatment groups balanced for: age, gender, polytrauma and unilateral or bilateral rib fractures. Other than the allocated intervention, both groups will be followed-up in the same way to exclude bias beyond procedures necessary for the allocation treatment. Randomisation will be to two groups:

1. ESP block plus multimodal analgesia (intervention)
2. Sham ESP block plus multimodal analgesia (control)

ESP block plus multimodal analgesia

Participants randomised to ESP block plus multimodal analgesia (intervention) will receive an US-guided ESP block story the vertebral transverse process corresponding to the mid-point of the consecutively fractured ribs on the side of pain. An initial fascial plane injection of 30ml of 0.25% levo-

For peer review only

bupivacaine will be placed, followed by catheter programmed-intermittent boluses of 15ml 0.125% levo-bupivacaine given 3 hourly with option patient or clinician top-up of 5ml up to every 1 hour. Participants allocated to intervention will additionally receive standard supportive care and multimodal analgesia according to British Orthopaedic Association 2016 guidelines. The site-specific adoption of multimodal analgesia regimes will be reviewed as part of the site feasibility.

Sham ESP block plus multimodal analgesia

Participants randomised to Sham ESP block plus multimodal analgesia (control) will receive a sham/placebo ultrasound-guided ESP block targeting the vertebral transverse process corresponding to the mid-point of the consecutively fractured ribs on the side of pain. A single 1ml subcutaneous injection of saline 0.9% will be made and a perineural catheter applied and affixed by skin-glue externally on the skin which will be dressed and connected to an infusion pump with patient-button which will remain turned off. Participants allocated to control will additionally receive standard supportive care and multimodal analgesia according to individual trial site protocol as per the intervention arm.

Participants in both arms will continue to receive multimodal analgesia as dictated by their usual clinical care team. Following ESPEAR enrolment, additional regional anaesthetic techniques (for example thoracic epidural insertion) will be undertaken at the discretion of the treating clinician, will be recorded in the trial CRF and will not lead to participant withdrawal.

Blinding

Participants will be blinded to group allocation. Placebo effects are known to play a significant role on pain perception and patient expectation of analgesic efficacy; therefore it is important that a definitive trial include a placebo arm. This pilot RCT will test this blinding effectiveness as part of the feasibility embedded qualitative process analysis. Anaesthetists siting the ESP block or Sham ESP block will not be blind to group allocation, since this is not technically possible. Outcome assessors will be blind to group allocation.

Outcome measures

Primary Feasibility Outcomes:

The primary feasibility outcomes, which will be measured to meet the objectives of this trial are:

For peer review only

- Recruitment rate. Defined as the number of eligible participants who consent to participate in the trial as a percentage of all eligible participants. This will be presented per centre per month and measured over the recruitment period (from randomisation of the first participant to randomisation of the final participant). The target recruitment rate is defined as recruitment of 50 participants from three recruiting centres, with each centre being open to recruitment for 12 months. This produces a mean trial target recruitment rate of 1.4 participants per centre per month.
- Retention rate. Defined as the proportion of randomised participants who complete 6-week follow-up with valid candidate clinical outcomes data (see below).
- Barriers and facilitators to recruitment and retention among participants and recruitment site staff (anaesthetists, allied health professionals, surgeons and research staff). This will be assessed in the embedded qualitative study.

Secondary Feasibility Outcomes

The secondary feasibility outcomes are as follows:

- Trial eligibility rate. Defined as the proportion of those patients screened who were eligible for enrolment in the trial.
- Trial consent rate. Defined as the proportion of eligible patients who provided written consent for inclusion in the trial.
- Willingness of anaesthetists to randomise patients to intervention or control and willingness of potential participants to randomisation. This will be achieved through qualitative evaluation, including scrutiny of screening logs, completion of an open-ended survey with healthcare staff and qualitative interviews with research staff conducted by the central research team.
- Causes of protocol violation. Causes will be identified from the Investigator Site File.

Secondary Clinical Outcomes

The following clinical outcomes are considered secondary outcomes of the trial, and will be measured to assess the relevance, completeness, and acceptability of these outcomes for use in a future definitive RCT:

For peer review only

- Static chest wall pain intensity. Measured on Short Form McGill Pain Questionnaire 2 (SF-MPQ-2) to describe the worst pain experienced by the patient between the following eight time points: 24 hours prior to receipt of the trial intervention (defined as trial baseline), then at 24, 48 and 72 hours. Scores will be described at each time interval in comparison to baseline and summed to provide a cumulative static chest wall pain score. The time at which each measure is taken will also be recorded.
- Functional (i.e. dynamic) chest wall pain intensity. Measured on a modified Functional Pain Scale (m-FPS) as the worst pain experienced by the patient during the following eight time points: 24 hours prior to receipt of the trial intervention (defined as trial baseline), then at 24, 48 and 72 hours. The time at which each measure is taken will also be recorded. Scores will be described at each time interval in comparison to baseline and summed to provide a cumulative functional chest wall pain score. The m-FPS consists of the following Likert-scaled responses: 0 = no pain; 1 = tolerable pain but able to perform vital capacity breath and effective cough; 2 = tolerable pain but prevents either vital capacity breath or effective cough; 3 = intolerable pain but can perform either vital capacity breath or effective cough; 4 = intolerable pain and unable to perform vital capacity breath or effective cough; 5 = intolerable and unable to verbally communicate due to pain.
- Forced vital capacity, forced expiratory volume in one second and peak cough flow (Spirometry). Measured by bed-side portable spirometry. Measured immediately prior to receipt of trial intervention (defined as trial baseline), then at the following time points following receipt of intervention: 3 hours, 6 hours, 9 hours, 12 hours, 24 hours, 48 hours and 72 hours.
- Cumulative non-opioid analgesic consumption. The administration of the non-opioid analgesics paracetamol and non-steroidal anti-inflammatories will be measured as total doses administered in the 24 hours prior to receipt of the trial intervention (defined as trial baseline), then at the following time points following receipt of the intervention: 24 hours, 48 hours and 72 hours.
- Cumulative opioid analgesic consumption. The administration of the opioid analgesics will be measured as total dose administered in the 24 hours prior to receipt of the trial intervention (defined as trial baseline), then at the following time points following receipt of the intervention: 24 hours, 48 hours and 72 hours. All doses will be converted to morphine-equivalents for analysis.

For peer review only

- Cumulative ketamine analgesic consumption. The administration of the ketamine will be measured as total dose administered in the 24 hours prior to receipt of the trial intervention (defined as trial baseline), then at the following time points following receipt of the intervention: 24 hours, 48 hours and 72 hours.
- Additional procedures of regional anaesthesia following ESP block. The administration of the following additional procedures of regional anaesthesia will be recorded in the 24 hours prior to receipt of the trial intervention (defined as trial baseline), then at the following time points following receipt of the intervention: 24 hours, 48 hours and 72 hours: intercostal, pleural, serratus plane, non-trial erector-spinae, paravertebral and epidural blockade.
- Opioid-related side-effects. The following opioid-related side-effects will be assessed immediately prior to receipt of trial intervention (defined as trial baseline) then at the following time points following receipt of intervention, 24 hours, 48 hours and 72 hours:
    - Constipation, defined as absence of bowel movement in the preceding 24 hour period.
    - Nausea or vomiting, scored on a 5-point scale (0 = no nausea or vomiting; 1 = mild nausea, no treatment required; 2 = nausea, anti-emetic administered; 3 = vomiting, anti-emetics administered; 4 = nausea or vomiting unresponsive to anti-emetic therapy).
    - Pruritis, scored on 11-point numerical rating scale.
    - Opioid-induced sedation, scored on Modified Observer's Assessment of Alertness/Sedation scale.
- Oxygen requirement. Measured as maximum flow rate of supplemental oxygen administered to participant immediately prior to receipt of trial intervention (defined as trial baseline), then at the following time points following receipt of intervention, 3, 6, 9, 12, 24 hours, 48 hours and 72 hours.
- Complications of regional anaesthesia. The following complications of regional anaesthesia will be assessed at 24 hours, 48 hours and 72 hours following receipt of intervention:
    - Treatment for local anaesthetic toxicity, defined as administration of intra-lipid therapy in the preceding 24 hour period.
    - Bleeding or infection at intervention insertion site.
    - Catheter dislodgement requiring re-sited intervention in preceding 24 hour period.
- Condition-specific outcome measure. Measured on Outcomes after Chest Trauma Score (OCTS) to describe severity of rib-related symptoms (domains include mobility, breathing, activities,

For peer review only

personal care, wellbeing and pain). The OCTS will be administered twice prior to receipt of trial intervention (defined as trial baseline) then at the following time points following receipt of intervention: 72 hours and 6 weeks.

- Diagnosis of pneumonia. Defined as administration of antibiotics for community- or hospital-acquired pneumonia assessed in the 24 hours prior to receipt of trial intervention (defined as trial baseline) then at the following time points following receipt of intervention, 24 hours, 48 hours and 72 hours and 6 weeks.
- Escalation of care to critical care. Defined as admission to Level 2 (HDU) or Level 3 (ICU) bed assessed in the 24 hours prior to receipt of trial intervention (defined as trial baseline) then at the following time points following receipt of intervention, 24 hours, 48 hours and 72 hours and 6 weeks.
- Length of hospital stay. Assessed 6 weeks following receipt of intervention.
- Quality of life measured on EQ-5D-5L. Assessed in the 24 hours prior to receipt of trial intervention (defined as trial baseline), then at 72 hours and 6 weeks following receipt of intervention.
- All-cause mortality. Assessed 6 weeks following receipt of intervention.

Sample size calculation

Formal sample size calculation is not appropriate for feasibility studies. Currently there is no single agreed method for sample size for a feasibility trial, but most authors propose a sample size between 24 and 60 depending on the study aims.[23,24] To answer our key objectives, we aim to recruit 50 participants, allowing estimation of recruitment and retention rates with a margin of error of less than 10%.

Statistical analysis

Data will be collected via REDCap database. Data analysis will primarily be descriptive to address the feasibility objectives of the trial. All analyses will be documented in a Statistical Analysis Plan which will be finalised prior to database lock. Feasibility outcomes will be estimated using descriptive statistics (with 95% confidence intervals [CI]) and will include screening rates, recruitment rates, follow-up rates, protocol adherence and amount of missing data for clinical outcomes. Key baseline characteristics

For peer review only

(age, sex) will be compared between trial participants and the ineligible and non-consenting patients, to ascertain adequacy of inclusion/exclusion criteria and likely generalisability of the trial to the required targeted population. Similarly, we will compare the key patient characteristics between those followed-up and those lost to follow-up and investigate how similar this is across the treatment arms to assess possible attrition bias in data collection. A baseline table will compare important demographic and clinical characteristics between the two treatment arms. It is not a primary objective of the feasibility trial to obtain definitive estimates of intervention effect on clinical outcomes and so the clinical outcomes will be analysed descriptively. Additionally, we will use appropriate regression method to estimate the likely range of intervention effects (point estimate and CIs) for key clinical outcomes adjusted for minimisation variables. Reporting of the study will be according to the CONSORT Statement: 2016 extension to randomised pilot and feasibility trials.[25]

For peer review only

ETHICS AND DISSEMINATION

The study was granted approval by the Oxford B Research Ethics Committee on 22 February 2022 (REC reference 22/SC/0005). Substantial amendments that require review by REC will not be implemented until the REC grants a favourable opinion for the trial. All personal identifiable information collected during the trial will be coded, depersonalised with unique codes for each patient. The trial will be compliant with the requirements of the General Data Protection Regulation 2018 and the Data Protection Act 2018. The chief investigator and principal investigators at participating sites will have access to the full dataset. Relevant anonymized patient level data will be made available on reasonable request. Day-to-day trial management will be provided by the Trial Management Group, who will meet at least once per month. Independent oversight of trial conduct will be provided by a Trial Steering Committee, attended by the trial Chief-investigators and methodologist, with three independent members with expertise in trial methodology and statistics, anaesthesia and trauma care.

A manuscript for a high-impact peer-reviewed journal will be prepared. Authorship will be determined in accordance with ICMJE guidelines,[26] and other contributors will be acknowledged. The results of this project will be disseminated to patients through local mechanisms at all participating centres.

For peer review only

REFERENCES

1. Ahn Y, Görlinger K, Alam HB, et al. Pain-associated respiratory failure in chest trauma. *Anesthesiol* 2013;118:701–8.

2. Sirmali M, Türüt H, Topçu S, et al. A comprehensive analysis of traumatic rib fractures: morbidity, mortality and management. *Journal of Cardio-Thoracic Surgery* 2003;24:133-138.

3. May L, Macadmed M, Hillermann C, et al. Rib fracture management. *Br J Anaesth Educ* 2016;16:26–32.

4. Kent R, Woods W, Bostrom O. Fatality risk and the presence of rib fractures. *Ann Adv Automot Med* 2008;52:73-82.

5. Holcomb JB, Mcmullin NR, Kozar RA, et al. Morbidity from rib fractures increases after age 45. *J Am Coll Surg* 2003;196:549–55.

6. London Major Trauma System: management of elderly major trauma patients. 2021. Available at: https://www.c4ts.qmul.ac.uk/downloads/2021-pan-london-major-trauma-system-management-of-older-trauma.-third-editionapril-2021.pdf. Accessed 04 March 2022.

7. Galvagno SM, Smith CE, Varon AJ, et al. Pain management for blunt thoracic trauma: a joint practice management guideline from the eastern association for the surgery of trauma and trauma anesthesiology society. *J Trauma Acute Care Surg* 2016;81:936-951.

8. Carrier FM, turgeon AF, nicole PC, et al. Effect of epidural analgesia in patients with traumatic rib fractures: a systematic review and meta-analysis of randomized controlled trials. *Can J Anesth* 2009;56:230–42.

9. Duch P, Møller MH. Epidural analgesia in patients with traumatic rib fractures: a systematic review of randomised controlled trials. *Acta Anaesthesiol Scand* 2015;59:698–709.

10. British Orthopaedic Association Standards for Trauma and Orthopaedics (BOASTS). Available at: Https://www.boa.ac.uk/standards-guidance/boasts.html. Accessed 04 March 2022.

11. El-Boghdadly K, Wiles MD. Regional anaesthesia for rib fractures: too many choices, too little evidence. *Anaesthesia* 2019;74:564–8.

12. Peek J, Smeeing DPJ, Hietbrink F, et al. Comparison of analgesic interventions for traumatic rib fractures: a systematic review and meta-analysis. *Eur J Trauma Emerg Surg* 2019;45:597-622.

For peer review only

13. Hewson DW, Bedforth NM, Hardman JG. Spinal cord injury arising in anaesthesia practice. Anaesthesia. 2018;73:43–50.

14. Beard l, Hillermann C, Beard E, et al. Multicenter longitudinal cross-sectional study comparing effectiveness of serratus anterior plane, paravertebral and thoracic epidural for the analgesia of multiple rib fractures. *Reg Anesth Pain Med* 2020;45:351–6.

15. Adhikary SD, Liu WM, Fuller E, et al. The effect of erector spinae plane block on respiratory and analgesic outcomes in multiple rib fractures: a retrospective cohort study. *Anaesthesia* 2019;74:585–93.

16. Forero M, Adhikary SD, Lopez H, et al. The erector spinae plane block. *Reg Anesth Pain Med* 2016;41:621–7.

17. Singh S, Choudhary NK, Lalin D, et al. Bilateral ultrasound-guided erector spinae plane block for postoperative analgesia in lumbar spine surgery: a randomized control trial. *J Neurosurg Anesthesiol* 2020;32:330–4.

18. Gürkan Y, Aksu C, Kuş A, et al. Ultrasound guided erector spinae plane block reduces postoperative opioid consumption following breast surgery: a randomized controlled study. *J Clin Anesth* 2018;50:65–8.

19. Ciftci B, Ekinci M, Celik EC, et al. Efficacy of an ultrasound-guided erector spinae plane block for postoperative analgesia management after video-assisted thoracic surgery: a prospective randomized study. *J Cardiothorac Vasc Anesth* 2020;34:444–9.

20. Macaire P, Ho N, Nguyen V, et al. Bilateral ultrasound-guided thoracic erector spinae plane blocks using a programmed intermittent bolus improve opioid-sparing postoperative analgesia in pediatric patients after open cardiac surgery: a randomized, double-blind, placebo-controlled trial. *Reg Anesth Pain Med* 2020;45:805–12.

21. El-Boghdadly K, Pawa A. The erector spinae plane block: plane and simple. *Anaesthesia* 2017;72:434–8.

22. Hamilton D, Manickam B. Erector spinae plane block for pain relief in rib fractures. *Br J Anaesth* 2017;118:474-5.

23. Julious SA. Sample size of 12 per group rule of thumb for a pilot study. *Pharm Stat* 2005;4:287–91.

24. Teare MD, dimairo M, shephard N, et al. Sample size requirements to estimate key design parameters from external pilot randomised controlled trials: a simulation study. *Trials* 2014;15:1–13.

For peer review only

25. Eldridge SM, Chan CL, Campbell MJ, et al. Consort 2010 statement: extension to randomised pilot and feasibility trials. *Pilot Feasibility Stud* 2016;Article Number 64. https://pilotfeasibilitystudies.biomedcentral.com/articles/10.1186/s40814-016-0105-8. Accessed 04 March 2022.

26. Recommendations for the conduct, reporting, editing and publication of scholarly work in medical journals. International Committee of Medical Journal Editors (ICMJE). Available at: http://www.icmje.org. Accessed 04 March 2022.

For peer review only

AUTHOR'S CONTRIBUTIONS

Conception of study: DWH, BO, NMB

Study design: DWH, JN, RO, BO, MLC, NMB

Ongoing trial management: All authors

Writing and final approval of protocol: All authors

FUNDING STATEMENT

This work was funded by the National Institute for Health Research (NIHR) (Research for Patient Benefit programme Call 42, [project reference: NIHR202195]). The views expressed are those of the authors and not necessarily those of the NIHR or the UK Department of Health and Social Care.

COMPETING INTERESTS

MLC is an NIHR Senior Investigator. The remaining authors declare no competing interests.

For peer review only

SPIRIT

STANDARD PROTOCOL ITEMS: RECOMMENDATIONS FOR INTERVENTIONAL TRIALS

Erector Spinae Plane blocks for the Early Analgesia of Rib fractures in trauma (ESPEAR): protocol for a multicentre pilot randomised controlled trial with feasibility and embedded qualitative assessment

Section/item	Item No	Description	ESPEAR PROTOCOL PAGE
**Administrative information**			
Title	1	Descriptive title identifying the study design, population, interventions, and, if applicable, trial acronym	1
Trial registration	2a	Trial identifier and registry name. If not yet registered, name of intended registry	1
	2b	All items from the World Health Organization Trial Registration Data Set	1
Protocol version	3	Date and version identifier	1
Funding	4	Sources and types of financial, material, and other support	20
Roles and responsibilities	5a	Names, affiliations, and roles of protocol contributors	1
	5b	Name and contact information for the trial sponsor	16
	5c	Role of study sponsor and funders, if any, in study design; collection, management, analysis, and interpretation of data; writing of the report; and the decision to submit the report for publication, including whether they will have ultimate authority over any of these activities	20

For peer review only

	5d	Composition, roles, and responsibilities of the coordinating centre, steering committee, endpoint adjudication committee, data management team, and other individuals or groups overseeing the trial, if applicable (see Item 21a for data monitoring committee)	16
**Introduction**			
Background and rationale	6a	Description of research question and justification for undertaking the trial, including summary of relevant studies (published and unpublished) examining benefits and harms for each intervention	4-6
	6b	Explanation for choice of comparators	10
Objectives	7	Specific objectives or hypotheses	8-9
Trial design	8	Description of trial design including type of trial (eg, parallel group, crossover, factorial, single group), allocation ratio, and framework (eg, superiority, equivalence, noninferiority, exploratory)	7
**Methods: Participants, interventions, and outcomes**			
Study setting	9	Description of study settings (eg, community clinic, academic hospital) and list of countries where data will be collected. Reference to where list of study sites can be obtained	7
Eligibility criteria	10	Inclusion and exclusion criteria for participants. If applicable, eligibility criteria for study centres and individuals who will perform the interventions (eg, surgeons, psychotherapists)	8
Interventions	11a	Interventions for each group with sufficient detail to allow replication, including how and when they will be administered	9-10
	11b	Criteria for discontinuing or modifying allocated interventions for a given trial participant (eg, drug dose change in response to harms, participant request, or improving/worsening disease)	10
	11c	Strategies to improve adherence to intervention protocols, and any procedures for monitoring adherence (eg, drug tablet return, laboratory tests)	Not provided
	11d	Relevant concomitant care and interventions that are permitted or prohibited during the trial	9-10

For peer review only - http://bmjopen.bmj.com/site/about/guidelines.xhtml

Outcomes	12	Primary, secondary, and other outcomes, including the specific measurement variable (eg, systolic blood pressure), analysis metric (eg, change from baseline, final value, time to event), method of aggregation (eg, median, proportion), and time point for each outcome. Explanation of the clinical relevance of chosen efficacy and harm outcomes is strongly recommended	10-14
Participant timeline	13	Time schedule of enrolment, interventions (including any run-ins and washouts), assessments, and visits for participants. A schematic diagram is highly recommended (see Figure)	10
Sample size	14	Estimated number of participants needed to achieve study objectives and how it was determined, including clinical and statistical assumptions supporting any sample size calculations	14
Recruitment	15	Strategies for achieving adequate participant enrolment to reach target sample size	Not provided
**Methods: Assignment of interventions (for controlled trials)**			
Allocation:			
Sequence generation	16a	Method of generating the allocation sequence (eg, computer-generated random numbers), and list of any factors for stratification. To reduce predictability of a random sequence, details of any planned restriction (eg, blocking) should be provided in a separate document that is unavailable to those who enrol participants or assign interventions	9
Allocation concealment mechanism	16b	Mechanism of implementing the allocation sequence (eg, central telephone; sequentially numbered, opaque, sealed envelopes), describing any steps to conceal the sequence until interventions are assigned	9
Implementation	16c	Who will generate the allocation sequence, who will enrol participants, and who will assign participants to interventions	9
Blinding (masking)	17a	Who will be blinded after assignment to interventions (eg, trial participants, care providers, outcome assessors, data analysts), and how	10

For peer review only - http://bmjopen.bmj.com/site/about/guidelines.xhtml

	17b	If blinded, circumstances under which unblinding is permissible, and procedure for revealing a participant's allocated intervention during the trial	Not provided
**Methods: Data collection, management, and analysis**			
Data collection methods	18a	Plans for assessment and collection of outcome, baseline, and other trial data, including any related processes to promote data quality (eg, duplicate measurements, training of assessors) and a description of study instruments (eg, questionnaires, laboratory tests) along with their reliability and validity, if known. Reference to where data collection forms can be found, if not in the protocol	14
	18b	Plans to promote participant retention and complete follow-up, including list of any outcome data to be collected for participants who discontinue or deviate from intervention protocols	Not provided
Data management	19	Plans for data entry, coding, security, and storage, including any related processes to promote data quality (eg, double data entry; range checks for data values). Reference to where details of data management procedures can be found, if not in the protocol	Not provided
Statistical methods	20a	Statistical methods for analysing primary and secondary outcomes. Reference to where other details of the statistical analysis plan can be found, if not in the protocol	14-15
	20b	Methods for any additional analyses (eg, subgroup and adjusted analyses)	14-15
	20c	Definition of analysis population relating to protocol non-adherence (eg, as randomised analysis), and any statistical methods to handle missing data (eg, multiple imputation)	14-15
**Methods: Monitoring**			
Data monitoring	21a	Composition of data monitoring committee (DMC); summary of its role and reporting structure; statement of whether it is independent from the sponsor and competing interests; and reference to where further details about its charter can be found, if not in the protocol. Alternatively, an explanation of why a DMC is not needed	16

For peer review only - http://bmjopen.bmj.com/site/about/guidelines.xhtml

	21b	Description of any interim analyses and stopping guidelines, including who will have access to these interim results and make the final decision to terminate the trial	14+16
Harms	22	Plans for collecting, assessing, reporting, and managing solicited and spontaneously reported adverse events and other unintended effects of trial interventions or trial conduct	16
Auditing	23	Frequency and procedures for auditing trial conduct, if any, and whether the process will be independent from investigators and the sponsor	16
**Ethics and dissemination**			
Research ethics approval	24	Plans for seeking research ethics committee/institutional review board (REC/IRB) approval	16
Protocol amendments	25	Plans for communicating important protocol modifications (eg, changes to eligibility criteria, outcomes, analyses) to relevant parties (eg, investigators, REC/IRBs, trial participants, trial registries, journals, regulators)	16
Consent or assent	26a	Who will obtain informed consent or assent from potential trial participants or authorised surrogates, and how (see Item 32)	16
	26b	Additional consent provisions for collection and use of participant data and biological specimens in ancillary studies, if applicable	N/A
Confidentiality	27	How personal information about potential and enrolled participants will be collected, shared, and maintained in order to protect confidentiality before, during, and after the trial	16
Declaration of interests	28	Financial and other competing interests for principal investigators for the overall trial and each study site	20
Access to data	29	Statement of who will have access to the final trial dataset, and disclosure of contractual agreements that limit such access for investigators	20
Ancillary and post-trial care	30	Provisions, if any, for ancillary and post-trial care, and for compensation to those who suffer harm from trial participation	Not provided

For peer review only

Dissemination policy	31a	Plans for investigators and sponsor to communicate trial results to participants, healthcare professionals, the public, and other relevant groups (eg, via publication, reporting in results databases, or other data sharing arrangements), including any publication restrictions	20
	31b	Authorship eligibility guidelines and any intended use of professional writers	20
	31c	Plans, if any, for granting public access to the full protocol, participant-level dataset, and statistical code	20
**Appendices**			
Informed consent materials	32	Model consent form and other related documentation given to participants and authorised surrogates	Supplementary material
Biological specimens	33	Plans for collection, laboratory evaluation, and storage of biological specimens for genetic or molecular analysis in the current trial and for future use in ancillary studies, if applicable	N/A

*It is strongly recommended that this checklist be read in conjunction with the SPIRIT 2013 Explanation & Elaboration for important clarification on the items. Amendments to the protocol should be tracked and dated. The SPIRIT checklist is copyrighted by the SPIRIT Group under the Creative Commons "Attribution-NonCommercial-NoDerivs 3.0 Unported" license.

For peer review only

BMJ Open

Erector Spinae Plane blocks for the Early Analgesia of Rib fractures in trauma (ESPEAR): protocol for a multicentre pilot randomised controlled trial with feasibility and embedded qualitative assessment

Journal:	*BMJ Open*
Manuscript ID	bmjopen-2022-062935.R1
Article Type:	Protocol
Date Submitted by the Author:	13-Aug-2022

Complete List of Authors:	Hewson, David; Nottingham University Hospitals NHS Trust, Department of Anaesthesia; University of Nottingham<br>Nightingale, Jessica; Nottingham University Hospitals NHS Trust, Trauma and Orthopaedics<br>Ogollah, Reuben; University of Nottingham University Park Campus, Nottingham Clinical Trials Unit<br>Ollivere, Benjamin; University of Nottingham, Trauma Outcomes Group<br>Costa, Matthew; Oxford University, Nuffield Department of Orthopaedics, Rheumatology, and Musculoskeletal Sciences<br>Craxford, Simon; University of Nottingham, Trauma and orthopaedics<br>Bates, Peter; Barts Health NHS Trust<br>Bedforth, Nigel; Nottingham University Hospitals NHS Trust, Department of Anaethesia

**Primary Subject Heading**:	Anaesthesia
Secondary Subject Heading:	Anaesthesia, Surgery, Emergency medicine
Keywords:	ANAESTHETICS, TRAUMA MANAGEMENT, PAIN MANAGEMENT, ACCIDENT & EMERGENCY MEDICINE

SCHOLARONE™
Manuscripts

Erector Spinae Plane blocks for the Early Analgesia of Rib fractures in trauma (ESPEAR): protocol for a multicentre pilot randomised controlled trial with feasibility and embedded qualitative assessment

David W Hewson,[1,2] Jessica Nightingale,[3] Reuben Ogollah,[4] Benjamin J Ollivere,[2,5] Matthew L Costa,[6] Simon Craxford,[5] Peter Bates,[7] Nigel M Bedforth.[1,2]

1. Department of Anaesthesia, Queen's Medical Centre, Nottingham University Hospitals NHS Trust, NG7 2UH, UK

2. Anaesthesia and Critical Care, Academic Unit of Injury, Recovery and Inflammation Sciences, School of Medicine, University of Nottingham, Nottingham NG7 2UH, UK

3. Department of Orthopaedic Surgery, Queen's Medical Centre, Nottingham University Hospitals NHS Trust, NG7 2UH, UK

4. Nottingham Clinical Trials Unit, Applied Health Research Building, University Park, University of Nottingham, NG7 2RD, UK

5. Orthopaedic Trauma Surgery, Academic Unit of Injury, Inflammation and Recovery Science, School of Medicine, University of Nottingham, Nottingham NG7 2UH, UK

6. Nuffield Department of Orthopaedics, Rheumatology and Musculoskeletal Sciences, University of Oxford, OX3 9DU, UK

7. Department of Orthopaedic Trauma, Royal London Hospital, Bart's Health, London, E1 1FR.

Corresponding author Dr David Hewson
david.hewson@nottingham.ac.uk
Department of Anaesthesia
C Floor, East Block
Queen's Medical Centre
Nottingham University Hospitals NHS Trust
Nottingham
NG7 2UH
United Kingdom

Word count 3859 words

For peer review only

ABSTRACT

Introduction

Patients with rib fractures commonly experience significant acute pain and are at risk of hypoxia, retained secretions, respiratory failure and death. Effective analgesia improves these outcomes. There is widespread variation in analgesic treatments given to patients including oral, intravenous and epidural routes of administration. Erector spinae place (ESP) blockade, a novel regional analgesic technique, may be effective, but high quality evidence is lacking.

Methods & analysis

To determine if a definitive trial of ESP blockade in rib fractures is possible, we are conducting a multicentre, randomised controlled pilot study with feasibility and qualitative assessment. Fifty adult patients with rib fractures will be randomised in a 1:1 ratio to ESP blockade with multimodal analgesia or placebo ESP blockade with multimodal analgesia. Participants and outcome assessors will be blinded. The primary feasibility outcomes are recruitment rate, retention rate and trial acceptability assessed by interview.

Ethics & dissemination

The study was approved by the Oxford B Research Ethics Committee on 22 February 2022 (REC reference 22/SC/0005). All participants will provide written consent. Trial results will be reported via peer-review and to grant funders.

Registration details

ISRCTN: 49307616. Protocol version 1.2 dated 07/02/22.

KEYWORDS

Rib fractures, chest trauma, regional anaesthesia, analgesia, erector spinae plane block.

For peer review only

STRENGTHS AND LIMITATIONS

- There is widespread variation in the care of patients following rib fractures. The clinical effectiveness of ESP blocks and catheters in this patient group is unclear.
- This is a feasibility study with piloting of candidate clinical outcome measures to determine if a definitive trial is feasible; the present work alone cannot answer whether ESP blocks are an effective analgesic modality for patients with rib fractures.
- The study will test if an analgesic placebo arm is an acceptable methodological feature for participants, clinicians and investigators.
- The study uses a programmed-intermittent bolus (PIB) regime for local anaesthetic delivery, but the ideal dose and method of delivery for ESP blocks remains unknown. The study will not answer this question.
- The study used a double-blind design (patients and outcome assessors) and the effectiveness of blinding will be determined by patient and staff interviews.

For peer review only

INTRODUCTION

The pain from rib fractures is often described by patients as the worst pain they have ever experienced. The major complication of this pain is that patients are unable to cough and breathe deeply, causing atelectasis, retained secretions, hypoxaemia, pneumonia and progressive respiratory failure. Deterioration may require mechanical ventilation on an intensive care unit and lead to death.[1,2] This morbidity and mortality is a direct result of severe pain and impaired gas exchange from underlying contused lung parenchyma and altered ventilatory mechanics from the bone injury.[3] The presence of rib fractures in trauma is associated with a significantly increased risk of death, regardless of other injuries, with an odds ratio of 1.4 (95% CI 1.3–1.6) for adults 18-45 years old and 2.5 (95% CI 2.3–2.8) for adults older than 64 years.[4] This injury is therefore particularly devastating for older adults who not only have a higher risk of death but are also likely to sustain rib fractures from less traumatic accidents (due to bone fragility), for example falling from standing height.[5]

A key objective in the multidisciplinary care of people with rib fractures is the assessment and treatment of pain to provide patient comfort and allow normal respiration and cough to minimise the risk of respiratory failure.[3,6] Alongside specialist physiotherapy and daily multidisciplinary review, good pain management is a vital element of early rib fracture care. Despite this, there is no agreement about the optimal pain relief to give patients. The literature on the use of the different analgesic techniques in rib fractures is inconclusive. Although national and international guidance recommends a multimodal approach in preference to opioid medications alone,[7] two meta-analyses concluded that the evidence to recommend any specific treatment modality is insufficient, and that there is no firm evidence for benefit or harm of one analgesic technique over another.[8,9] This leaves clinicians unsure of which analgesic techniques to use. National UK guidance specifies protocolised analgesic regimes as a standard of care for every patient with multiple rib fractures.[10] However the paucity of evidence meant that this guidance could not recommend which analgesic modality (epidural, peripheral nerve blocks or opioid) should be used in which clinical circumstances. Most patients with rib fractures are given a combination of analgesic drugs like paracetamol, non-steroidal anti-inflammatories, opioids and ketamine to help them cope with severe pain; these are the cornerstones of multimodal analgesia in this setting. Medication side effects (including nausea, pruritus, hallucinations, constipation, renal failure and respiratory depression) significantly limit their use. Some patients receive thoracic epidural analgesia (TEA) and some receive other forms of regional

For peer review only

anaesthesia nerve blocks, but the delivery of these interventions by pain-specialist anaesthetists is driven more by local expertise and experience than by high quality evidence.[11]

Regional anaesthesia (including nerve blocks) are clinically useful following rib fractures due to their opioid sparing effect (therefore reducing serious drug related side-effects) and the superior dynamic pain relief they provide. Traditional techniques to block the thoracic nerve supply to the ribs include thoracic epidural analgesia (TEA), paravertebral blockade and intercostal blockade. Systematic review and meta-analysis of these techniques suggested that TEA provides good pain relief, however this benefit does not translate into superior outcomes such as occurrence of pulmonary complications and length of time spent in hospital, intensive care or requiring mechanical ventilation.[12] Unfortunately, TEA has a significant failure rate and is also associated with common and potentially catastrophic complications [13] leading to permanent paralysis, and is therefore contraindicated in approximately one fifth of people with significant injuries. TEA is a complex intervention to perform, practiced by a small and reducing number of anaesthetists nationally and is not available equitably to patients. Even within a single hospital the care delivered varies depending on the time of day and availability of staff to perform such a complex analgesic technique. Only an estimated 9.9-18.4% of patients receive TEA for rib fracture pain.[14]

The erector spinae plane (ESP) block is a regional anaesthetic technique involving the infiltration and infusion of local anaesthetic along fascial planes containing dorsal and ventral rami of thoracic spinal nerves supplying the chest wall.[15] The injection is performed away from the spinal cord (thereby avoiding the complications of TEA). ESP blocks were first described in 2016 [16] and have demonstrated analgesic efficacy for patients on enhanced recovery after surgery protocols (ERAS) following spinal,[17] breast,[18] thoracic [19] and cardiac surgery.[20] In these post-operative acute pain settings, ESP blocks have been shown to reduce patient-reported pain scores and opioid consumption significantly in the early post-operative period compared to multimodal analgesia regimes alone. However, the role of ESP blocks in the management of acute rib fracture pain is currently uncertain.[21] There are no experimental pragmatic multi- centre trials published in this setting, however single-centre cohort data demonstrates ESP blocks provide effective pain relief and improve respiratory function when added to multimodal analgesia in patients with rib fractures.[15,22] Higher quality clinical evidence is urgently needed to guide clinicians on whether the ESP block is a suitable addition to current multimodal analgesia in patients with rib fractures. A definitive trial on this

For peer review only

topic would promote evidence-based practice in rib fracture management and reduce unnecessary variation in clinical practice across UK trauma centres. However there is currently not enough evidence on the effectiveness and acceptability of ESP blocks for rib fractures to undertake a definitive randomised controlled trial (RCT).

The aim of this study is therefore to determine if it is feasible to undertake a definitive RCT to establish if ESP blocks are a clinically effective early treatment for acute pain in patients hospitalised with rib fractures. Formal hypothesis testing for effectiveness or efficacy is not undertaken in feasibility studies. The aim of this trial is not to assess effectiveness or efficacy but to determine feasibility of progression to a definitive RCT.

For peer review only

METHODS AND ANALYSIS

Objectives

This study has the primary objective of determining whether it is feasible to undertake a definitive RCT to establish if ESP blocks are a clinically effective early treatment for acute pain in patients hospitalised with rib fractures. Our primary objectives are to determine:

- Trial recruitment rate.
- Trial retention rate.
- Barriers and facilitators to recruitment and retention among participants and recruitment site staff (anaesthetists, allied health professionals, surgeons and research staff) with regard to the acceptability of the trial intervention.

Secondary trial objectives are:

- To determine the willingness of anaesthetists to randomise patients to intervention or control and willingness of potential participants to randomisation.
- To identify causes of protocol violation and trial withdrawal.
- To assess the completeness of data arising from the trial.
- To assess the fidelity of the trial intervention in terms of ESP catheter dislodgement, blockage or other technical failure.
- To assess the acceptability of the intervention to participants.
- To describe complications of the intervention.
- To pilot the collection of candidate outcome measures for a future definitive trial.
- To determine preliminary indicators of effectiveness as measured by candidate clinical outcome measures.

Patient and Public involvement

The study question builds on previous qualitative work undertaken to validate a patient-derived recovery scale. The scale was developed following interviews with 50 patients and health professionals, with subsequent validation in a 250-patient study. This work characterised the

For peer review only

experience of pain and breathing difficulties following rib fracture; identifying management of pain as a research priority for this patient population. The outcome scale developed through this study will be used as an outcome measure in this trial, to help capture patient-centered outcomes. Specifically for this study we have facilitated virtual focus groups with patients who have previously sustained rib fractures and were admitted to Nottingham University Hospitals NHS Trust. The groups discussed the following aspects: the question and study design; recruitment and consent; follow-up data collection; acceptability of blinding and preferred outcome measures. There was strong support for this study; with individuals acknowledging that pain management of non-operatively managed injuries was an important but often overlooked area of their care. A new regional anaesthetic technique was perceived to be valuable as a treatment option or adjunct since participants said they would be keen to avoid the side-effects associated with oral and intravenous analgesia. The ESP block was perceived by focus group members as less invasive than an epidural. The inclusion of a sham intervention was discussed and deemed acceptable given the integrity of the research. The proposed outcome measures were reviewed by participants and were felt to be comprehensive. They valued the addition of embedded qualitative work within the study to allow for holistic feedback about study acceptability for patients and staff.

Population and setting

The target population is patients newly admitted to the MTC with one or more new rib fractures who can receive the trial intervention within 12 hours of admission to hospital. Participants will be recruited via their usual clinical care teams (emergency department, major trauma and/or acute pain services), who will notify study investigators of a potentially eligible participant for screening and recruitment purposes.

Inclusion criteria:

- Age ≥ 18
- New admission to major trauma centre and can receive trial intervention within 12 hours of admission
- Mechanism of injury blunt thoracic trauma
- Radiographic evidence of 1 or more new traumatic rib fractures

For peer review only

- Moderate or severe unilateral acute pain (defined as 11-point numerical rating scale (NRS) pain >4 when patient performing vital capacity breath or effective cough) at time of enrolment. Patients may have bilateral fractures, but pain must be unilateral.

Exclusion Criteria:

- Patient refusal or inability to give informed written consent for any reason
- Thoracic injury requiring emergent operative or interventional radiology management
- Allergy to local anaesthetic
- Infection at site of ESP block
- Actual or estimated total body weight ≤ 50 kg thereby precluding safe dosing of local anaesthetic for ESP block

Interventions and blinding

Following written consent, participant randomisation will be performed to a 1:1 ratio using a web-based automated computer-generated minimisation algorithm with treatment groups balanced for: age, gender, polytrauma and unilateral or bilateral rib fractures. Other than the allocated intervention, both groups will be followed-up in the same way to exclude bias beyond procedures necessary for the allocation treatment. Randomisation will be to two groups:

1. ESP block and catheter plus multimodal analgesia (intervention)
2. Sham ESP block and catheter plus multimodal analgesia (control)

ESP block plus multimodal analgesia

Participants randomised to ESP block plus multimodal analgesia (intervention) will receive an US-guided ESP block and catheter targeting the vertebral transverse process corresponding to the mid-point of the consecutively fractured ribs on the side of pain. An initial fascial plane injection of 30ml of 0.25% levo-bupivacaine will be placed, followed by catheter-delivered programmed-intermittent boluses of 15ml 0.125% levo-bupivacaine given every 3 hours with optional patient or clinician bolus of 5ml every 1 hour.

Participants allocated to intervention will additionally receive standard supportive care and multimodal analgesia according to British Orthopaedic Association 2016 guidelines. The site-specific adoption of multimodal analgesia regimes will be reviewed as part of the site feasibility.

For peer review only

Sham ESP block plus multimodal analgesia

Participants randomised to Sham ESP block plus multimodal analgesia (control) will receive a sham/placebo ultrasound-guided ESP block and catheter targeting the vertebral transverse process corresponding to the mid-point of the consecutively fractured ribs on the side of pain. A single 1ml subcutaneous injection of saline 0.9% will be made and a perineural catheter applied and affixed by skin-glue externally on the skin which will be dressed and connected to an infusion pump with patient-button which will remain turned off. Participants allocated to control will additionally receive standard supportive care and multimodal analgesia according to individual trial site protocol as per the intervention arm.

Participants in both arms will continue to receive multimodal analgesia as dictated by their usual clinical care team. Following ESPEAR enrolment, additional regional anaesthetic techniques (for example thoracic epidural insertion) will be undertaken at the discretion of the treating clinician, will be recorded in the trial CRF and will not lead to participant withdrawal.

Blinding

Participants will be blinded to group allocation. Placebo effects are known to play a significant role on pain perception and patient expectation of analgesic efficacy; therefore it is important that a definitive trial include a placebo arm. This pilot RCT will test this blinding effectiveness as part of the feasibility embedded qualitative process analysis. Anaesthetists siting the ESP block or Sham ESP block will not be blind to group allocation, since this is not technically possible. Outcome assessors will be blind to group allocation. Blinding will be achieved by the infusion devices in both arms being stored in a black carry-case during the infusion.

Outcome measures

Primary Feasibility Outcomes:

The primary feasibility outcomes, which will be measured to meet the objectives of this trial are:

- Recruitment rate. Defined as the number of eligible participants who consent to participate in the trial as a percentage of all eligible participants. This will be presented per centre per month and measured over the recruitment period (from randomisation of the first participant to

For peer review only

randomisation of the final participant). The target recruitment rate is defined as recruitment of 50 participants from three recruiting centres, with each centre being open to recruitment for 12 months. This produces a mean trial target recruitment rate of 1.4 participants per centre per month.

- Retention rate. Defined as the proportion of randomised participants who complete 6-week follow-up with valid candidate clinical outcomes data (see below).
- Barriers and facilitators to recruitment and retention among participants and recruitment site staff (anaesthetists, allied health professionals, surgeons and research staff). This will be assessed in the embedded qualitative study.

Secondary Feasibility Outcomes

The secondary feasibility outcomes are as follows:

- Trial eligibility rate. Defined as the proportion of those patients screened who were eligible for enrolment in the trial.
- Trial consent rate. Defined as the proportion of eligible patients who provided written consent for inclusion in the trial.
- Willingness of anaesthetists to randomise patients to intervention or control and willingness of potential participants to randomisation. This will be achieved through qualitative evaluation, including scrutiny of screening logs, completion of an open-ended survey with healthcare staff and qualitative interviews with research staff conducted by the central research team.
- Causes of protocol violation. Causes will be identified from the Investigator Site File.

Secondary Clinical Outcomes

The following clinical outcomes are considered secondary outcomes of the trial, and will be measured to assess the relevance, completeness, and acceptability of these outcomes for use in a future definitive RCT:

- Static chest wall pain intensity. Measured on Short Form McGill Pain Questionnaire 2 (SF-MPQ-2) to describe the worst pain experienced by the patient between the following eight time points: 24 hours prior to receipt of the trial intervention (defined as trial baseline), then at 24, 48 and 72 hours. Scores will be described at each time interval in comparison to baseline and

For peer review only

summed to provide a cumulative static chest wall pain score. The time at which each measure is taken will also be recorded.

- Functional (i.e. dynamic) chest wall pain intensity. Measured on a modified Functional Pain Scale (m-FPS) as the worst pain experienced by the patient during the following eight time points: 24 hours prior to receipt of the trial intervention (defined as trial baseline), then at 24, 48 and 72 hours. The time at which each measure is taken will also be recorded. Scores will be described at each time interval in comparison to baseline and summed to provide a cumulative functional chest wall pain score. The m-FPS consists of the following Likert-scaled responses: 0 = no pain; 1 = tolerable pain but able to perform vital capacity breath and effective cough; 2 = tolerable pain but prevents either vital capacity breath or effective cough; 3 = intolerable pain but can perform either vital capacity breath or effective cough; 4 = intolerable pain and unable to perform vital capacity breath or effective cough; 5 = intolerable and unable to verbally communicate due to pain.
- Forced vital capacity, forced expiratory volume in one second and peak cough flow (Spirometry). Measured by bed-side portable spirometry. Measured immediately prior to receipt of trial intervention (defined as trial baseline), then at the following time points following receipt of intervention: 3 hours, 6 hours, 9 hours, 12 hours, 24 hours, 48 hours and 72 hours.
- Cumulative non-opioid analgesic consumption. The administration of the non-opioid analgesics paracetamol and non-steroidal anti-inflammatories will be measured as total doses administered in the 24 hours prior to receipt of the trial intervention (defined as trial baseline), then at the following time points following receipt of the intervention: 24 hours, 48 hours and 72 hours.
- Cumulative opioid analgesic consumption. The administration of the opioid analgesics will be measured as total dose administered in the 24 hours prior to receipt of the trial intervention (defined as trial baseline), then at the following time points following receipt of the intervention: 24 hours, 48 hours and 72 hours. All doses will be converted to morphine-equivalents for analysis.
- Cumulative ketamine analgesic consumption. The administration of the ketamine will be measured as total dose administered in the 24 hours prior to receipt of the trial intervention (defined as trial baseline), then at the following time points following receipt of the intervention: 24 hours, 48 hours and 72 hours.

For peer review only

- Additional procedures of regional anaesthesia following ESP block. The administration of the following additional procedures of regional anaesthesia will be recorded in the 24 hours prior to receipt of the trial intervention (defined as trial baseline), then at the following time points following receipt of the intervention: 24 hours, 48 hours and 72 hours: intercostal, pleural, serratus plane, non-trial erector-spinae, paravertebral and epidural blockade.
- Opioid-related side-effects. The following opioid-related side-effects will be assessed immediately prior to receipt of trial intervention (defined as trial baseline) then at the following time points following receipt of intervention, 24 hours, 48 hours and 72 hours:
    - Constipation, defined as absence of bowel movement in the preceding 24 hour period.
    - Nausea or vomiting, scored on a 5-point scale (0 = no nausea or vomiting; 1 = mild nausea, no treatment required; 2 = nausea, anti-emetic administered; 3 = vomiting, anti-emetics administered; 4 = nausea or vomiting unresponsive to anti-emetic therapy).
    - Pruritis, scored on 11-point numerical rating scale.
    - Opioid-induced sedation, scored on Modified Observer's Assessment of Alertness/Sedation scale.
- Oxygen requirement. Measured as maximum flow rate of supplemental oxygen administered to participant immediately prior to receipt of trial intervention (defined as trial baseline), then at the following time points following receipt of intervention, 3, 6, 9, 12, 24 hours, 48 hours and 72 hours.
- Complications of regional anaesthesia. The following complications of regional anaesthesia will be assessed at 24 hours, 48 hours and 72 hours following receipt of intervention:
    - Treatment for local anaesthetic toxicity, defined as administration of intra-lipid therapy in the preceding 24 hour period.
    - Bleeding or infection at intervention insertion site.
    - Catheter dislodgement requiring re-sited intervention in preceding 24 hour period.
- Condition-specific outcome measure. Measured on Outcomes after Chest Trauma Score (OCTS) to describe severity of rib-related symptoms (domains include mobility, breathing, activities, personal care, wellbeing and pain). The OCTS will be administered twice prior to receipt of trial intervention (defined as trial baseline) then at the following time points following receipt of intervention: 72 hours and 6 weeks.

For peer review only

- Diagnosis of pneumonia. Defined as administration of antibiotics for community- or hospital-acquired pneumonia assessed in the 24 hours prior to receipt of trial intervention (defined as trial baseline) then at the following time points following receipt of intervention, 24 hours, 48 hours and 72 hours and 6 weeks.
- Escalation of care to critical care. Defined as admission to Level 2 (HDU) or Level 3 (ICU) bed assessed in the 24 hours prior to receipt of trial intervention (defined as trial baseline) then at the following time points following receipt of intervention, 24 hours, 48 hours and 72 hours and 6 weeks.
- Length of hospital stay. Assessed 6 weeks following receipt of intervention.
- Quality of life measured on EQ-5D-5L. Assessed in the 24 hours prior to receipt of trial intervention (defined as trial baseline), then at 72 hours and 6 weeks following receipt of intervention.
- All-cause mortality. Assessed 6 weeks following receipt of intervention.

Sample size calculation

Formal sample size calculation is not appropriate for feasibility studies. Currently there is no single agreed method for sample size for a feasibility trial, but most authors propose a sample size between 24 and 60 depending on the study aims.[23,24] To answer our key objectives, we aim to recruit 50 participants, allowing estimation of recruitment and retention rates with a margin of error of less than 10%.

Statistical analysis

Data will be collected via REDCap database. Data analysis will primarily be descriptive to address the feasibility objectives of the trial. All analyses will be documented in a Statistical Analysis Plan which will be finalised prior to database lock. Feasibility outcomes will be estimated using descriptive statistics (with 95% confidence intervals [CI]) and will include screening rates, recruitment rates, follow-up rates, protocol adherence and amount of missing data for clinical outcomes. Key baseline characteristics (age, sex) will be compared between trial participants and the ineligible and non-consenting patients, to ascertain adequacy of inclusion/exclusion criteria and likely generalisability of the trial to the required targeted population. Similarly, we will compare the key patient characteristics between those

For peer review only

followed-up and those lost to follow-up and investigate how similar this is across the treatment arms to assess possible attrition bias in data collection. A baseline table will compare important demographic and clinical characteristics between the two treatment arms. It is not a primary objective of the feasibility trial to obtain definitive estimates of intervention effect on clinical outcomes and so the clinical outcomes will be analysed descriptively. Additionally, we will use appropriate regression method to estimate the likely range of intervention effects (point estimate and CIs) for key clinical outcomes adjusted for minimisation variables. Reporting of the study will be according to the CONSORT Statement: 2016 extension to randomised pilot and feasibility trials.[25]

For peer review only

ETHICS AND DISSEMINATION

The study was granted approval by the Oxford B Research Ethics Committee on 22 February 2022 (REC reference 22/SC/0005). Substantial amendments that require review by REC will not be implemented until the REC grants a favourable opinion for the trial. All personal identifiable information collected during the trial will be coded, depersonalised with unique codes for each patient. The trial will be compliant with the requirements of the General Data Protection Regulation 2018 and the Data Protection Act 2018. The chief investigator and principal investigators at participating sites will have access to the full dataset. Relevant anonymized patient level data will be made available on reasonable request. Day-to-day trial management will be provided by the Trial Management Group, who will meet at least once per month. Independent oversight of trial conduct will be provided by a Trial Steering Committee, attended by the trial Chief-investigators and methodologist, with three independent members with expertise in trial methodology and statistics, anaesthesia and trauma care.

A manuscript for a high-impact peer-reviewed journal will be prepared. Authorship will be determined in accordance with ICMJE guidelines,[26] and other contributors will be acknowledged. The results of this project will be disseminated to patients through local mechanisms at all participating centres.

For peer review only

AUTHOR'S CONTRIBUTIONS

DWH conceived the study, refined the study design, is responsible for ongoing trial management and wrote and approved this protocol. JN refined the study design, is responsible for ongoing trial management and wrote and approved this protocol. RO refined the study design, is responsible for ongoing trial management and wrote and approved this protocol. BJO conceived the study, is responsible for ongoing trial management and wrote and approved this protocol. NMB conceived the study, refined the study design, is responsible for ongoing trial management and wrote and approved this protocol. MLC refined the study design, is responsible for ongoing trial management and wrote and approved this protocol. SC and PB are responsible for ongoing trial management and wrote and approved this protocol.

For peer review only

FUNDING STATEMENT

This work was funded by the National Institute for Health Research (NIHR) (Research for Patient Benefit programme Call 42, [project reference: NIHR202195]). The views expressed are those of the authors and not necessarily those of the NIHR or the UK Department of Health and Social Care.

COMPETING INTERESTS

MLC is an NIHR Senior Investigator. The remaining authors declare no competing interests.

REFERENCES

1. Ahn Y, Görlinger K, Alam HB, et al. Pain-associated respiratory failure in chest trauma. *Anesthesiol* 2013;118:701–8.

2. Sirmali M, Türüt H, Topçu S, et al. A comprehensive analysis of traumatic rib fractures: morbidity, mortality and management. *Journal of Cardio-Thoracic Surgery* 2003;24:133-138.

3. May L, Macadmed M, Hillermann C, et al. Rib fracture management. *Br J Anaesth Educ* 2016;16:26–32.

4. Kent R, Woods W, Bostrom O. Fatality risk and the presence of rib fractures. *Ann Adv Automot Med* 2008;52:73-82.

5. Holcomb JB, Mcmullin NR, Kozar RA, et al. Morbidity from rib fractures increases after age 45. *J Am Coll Surg* 2003;196:549–55.

6. London Major Trauma System: management of elderly major trauma patients. 2021. Available at: https://www.c4ts.qmul.ac.uk/downloads/2021-pan-london-major-trauma-system-management-of-older-trauma.-third-editionapril-2021.pdf. Accessed 04 March 2022.

7. Galvagno SM, Smith CE, Varon AJ, et al. Pain management for blunt thoracic trauma: a joint practice management guideline from the eastern association for the surgery of trauma and trauma anesthesiology society. *J Trauma Acute Care Surg* 2016;81:936-951.

8. Carrier FM, turgeon AF, nicole PC, et al. Effect of epidural analgesia in patients with traumatic rib fractures: a systematic review and meta-analysis of randomized controlled trials. *Can J Anesth* 2009;56:230–42.

9. Duch P, Møller MH. Epidural analgesia in patients with traumatic rib fractures: a systematic review of randomised controlled trials. *Acta Anaesthesiol Scand* 2015;59:698–709.

10. British Orthopaedic Association Standards for Trauma and Orthopaedics (BOASTS). Available at: Https://www.boa.ac.uk/standards-guidance/boasts.html. Accessed 04 March 2022.

11. El-Boghdadly K, Wiles MD. Regional anaesthesia for rib fractures: too many choices, too little evidence. *Anaesthesia* 2019;74:564–8.

12. Peek J, Smeeing DPJ, Hietbrink F, et al. Comparison of analgesic interventions for traumatic rib fractures: a systematic review and meta-analysis. *Eur J Trauma Emerg Surg* 2019;45:597-622.

For peer review only

13. Hewson DW, Bedforth NM, Hardman JG. Spinal cord injury arising in anaesthesia practice. Anaesthesia. 2018;73:43–50.

14. Beard l, Hillermann C, Beard E, et al. Multicenter longitudinal cross-sectional study comparing effectiveness of serratus anterior plane, paravertebral and thoracic epidural for the analgesia of multiple rib fractures. *Reg Anesth Pain Med* 2020;45:351–6.

15. Adhikary SD, Liu WM, Fuller E, et al. The effect of erector spinae plane block on respiratory and analgesic outcomes in multiple rib fractures: a retrospective cohort study. *Anaesthesia* 2019;74:585–93.

16. Forero M, Adhikary SD, Lopez H, et al. The erector spinae plane block. *Reg Anesth Pain Med* 2016;41:621–7.

17. Singh S, Choudhary NK, Lalin D, et al. Bilateral ultrasound-guided erector spinae plane block for postoperative analgesia in lumbar spine surgery: a randomized control trial. *J Neurosurg Anesthesiol* 2020;32:330–4.

18. Gürkan Y, Aksu C, Kuş A, et al. Ultrasound guided erector spinae plane block reduces postoperative opioid consumption following breast surgery: a randomized controlled study. *J Clin Anesth* 2018;50:65–8.

19. Ciftci B, Ekinci M, Celik EC, et al. Efficacy of an ultrasound-guided erector spinae plane block for postoperative analgesia management after video-assisted thoracic surgery: a prospective randomized study. *J Cardiothorac Vasc Anesth* 2020;34:444–9.

20. Macaire P, Ho N, Nguyen V, et al. Bilateral ultrasound-guided thoracic erector spinae plane blocks using a programmed intermittent bolus improve opioid-sparing postoperative analgesia in pediatric patients after open cardiac surgery: a randomized, double-blind, placebo-controlled trial. *Reg Anesth Pain Med* 2020;45:805–12.

21. El-Boghdadly K, Pawa A. The erector spinae plane block: plane and simple. *Anaesthesia* 2017;72:434–8.

22. Hamilton D, Manickam B. Erector spinae plane block for pain relief in rib fractures. *Br J Anaesth* 2017;118:474-5.

23. Julious SA. Sample size of 12 per group rule of thumb for a pilot study. *Pharm Stat* 2005;4:287–91.

24. Teare MD, dimairo M, shephard N, et al. Sample size requirements to estimate key design parameters from external pilot randomised controlled trials: a simulation study. *Trials* 2014;15:1–13.

For peer review only

25. Eldridge SM, Chan CL, Campbell MJ, et al. Consort 2010 statement: extension to randomised pilot and feasibility trials. *Pilot Feasibility Stud* 2016;Article Number 64. https://pilotfeasibilitystudies.biomedcentral.com/articles/10.1186/s40814-016-0105-8. Accessed 04 March 2022.

26. Recommendations for the conduct, reporting, editing and publication of scholarly work in medical journals. International Committee of Medical Journal Editors (ICMJE). Available at: http://www.icmje.org. Accessed 04 March 2022.

For peer review only

For peer review only

SPIRIT
Standard Protocol Items: Recommendations for Interventional Trials

Erector Spinae Plane blocks for the Early Analgesia of Rib fractures in trauma (ESPEAR): protocol for a multicentre pilot randomised controlled trial with feasibility and embedded qualitative assessment

Section/item	Item No	Description	ESPEAR PROTOCOL PAGE
**Administrative information**			
Title	1	Descriptive title identifying the study design, population, interventions, and, if applicable, trial acronym	1
Trial registration	2a	Trial identifier and registry name. If not yet registered, name of intended registry	1
	2b	All items from the World Health Organization Trial Registration Data Set	1
Protocol version	3	Date and version identifier	1
Funding	4	Sources and types of financial, material, and other support	20
Roles and responsibilities	5a	Names, affiliations, and roles of protocol contributors	1
	5b	Name and contact information for the trial sponsor	16
	5c	Role of study sponsor and funders, if any, in study design; collection, management, analysis, and interpretation of data; writing of the report; and the decision to submit the report for publication, including whether they will have ultimate authority over any of these activities	20

For peer review only

	5d	Composition, roles, and responsibilities of the coordinating centre, steering committee, endpoint adjudication committee, data management team, and other individuals or groups overseeing the trial, if applicable (see Item 21a for data monitoring committee)	16
**Introduction**			
Background and rationale	6a	Description of research question and justification for undertaking the trial, including summary of relevant studies (published and unpublished) examining benefits and harms for each intervention	4-6
	6b	Explanation for choice of comparators	10
Objectives	7	Specific objectives or hypotheses	8-9
Trial design	8	Description of trial design including type of trial (eg, parallel group, crossover, factorial, single group), allocation ratio, and framework (eg, superiority, equivalence, noninferiority, exploratory)	7
**Methods: Participants, interventions, and outcomes**			
Study setting	9	Description of study settings (eg, community clinic, academic hospital) and list of countries where data will be collected. Reference to where list of study sites can be obtained	7
Eligibility criteria	10	Inclusion and exclusion criteria for participants. If applicable, eligibility criteria for study centres and individuals who will perform the interventions (eg, surgeons, psychotherapists)	8
Interventions	11a	Interventions for each group with sufficient detail to allow replication, including how and when they will be administered	9-10
	11b	Criteria for discontinuing or modifying allocated interventions for a given trial participant (eg, drug dose change in response to harms, participant request, or improving/worsening disease)	10
	11c	Strategies to improve adherence to intervention protocols, and any procedures for monitoring adherence (eg, drug tablet return, laboratory tests)	Not provided
	11d	Relevant concomitant care and interventions that are permitted or prohibited during the trial	9-10

For peer review only

For peer review only - http://bmjopen.bmj.com/site/about/guidelines.xhtml

Outcomes	12	Primary, secondary, and other outcomes, including the specific measurement variable (eg, systolic blood pressure), analysis metric (eg, change from baseline, final value, time to event), method of aggregation (eg, median, proportion), and time point for each outcome. Explanation of the clinical relevance of chosen efficacy and harm outcomes is strongly recommended	10-14
Participant timeline	13	Time schedule of enrolment, interventions (including any run-ins and washouts), assessments, and visits for participants. A schematic diagram is highly recommended (see Figure)	10
Sample size	14	Estimated number of participants needed to achieve study objectives and how it was determined, including clinical and statistical assumptions supporting any sample size calculations	14
Recruitment	15	Strategies for achieving adequate participant enrolment to reach target sample size	Not provided
**Methods: Assignment of interventions (for controlled trials)**			
Allocation:			
Sequence generation	16a	Method of generating the allocation sequence (eg, computer-generated random numbers), and list of any factors for stratification. To reduce predictability of a random sequence, details of any planned restriction (eg, blocking) should be provided in a separate document that is unavailable to those who enrol participants or assign interventions	9
Allocation concealment mechanism	16b	Mechanism of implementing the allocation sequence (eg, central telephone; sequentially numbered, opaque, sealed envelopes), describing any steps to conceal the sequence until interventions are assigned	9
Implementation	16c	Who will generate the allocation sequence, who will enrol participants, and who will assign participants to interventions	9
Blinding (masking)	17a	Who will be blinded after assignment to interventions (eg, trial participants, care providers, outcome assessors, data analysts), and how	10

	17b	If blinded, circumstances under which unblinding is permissible, and procedure for revealing a participant's allocated intervention during the trial	Not provided
**Methods: Data collection, management, and analysis**			
Data collection methods	18a	Plans for assessment and collection of outcome, baseline, and other trial data, including any related processes to promote data quality (eg, duplicate measurements, training of assessors) and a description of study instruments (eg, questionnaires, laboratory tests) along with their reliability and validity, if known. Reference to where data collection forms can be found, if not in the protocol	14
	18b	Plans to promote participant retention and complete follow-up, including list of any outcome data to be collected for participants who discontinue or deviate from intervention protocols	Not provided
Data management	19	Plans for data entry, coding, security, and storage, including any related processes to promote data quality (eg, double data entry; range checks for data values). Reference to where details of data management procedures can be found, if not in the protocol	Not provided
Statistical methods	20a	Statistical methods for analysing primary and secondary outcomes. Reference to where other details of the statistical analysis plan can be found, if not in the protocol	14-15
	20b	Methods for any additional analyses (eg, subgroup and adjusted analyses)	14-15
	20c	Definition of analysis population relating to protocol non-adherence (eg, as randomised analysis), and any statistical methods to handle missing data (eg, multiple imputation)	14-15
**Methods: Monitoring**			
Data monitoring	21a	Composition of data monitoring committee (DMC); summary of its role and reporting structure; statement of whether it is independent from the sponsor and competing interests; and reference to where further details about its charter can be found, if not in the protocol. Alternatively, an explanation of why a DMC is not needed	16

	21b	Description of any interim analyses and stopping guidelines, including who will have access to these interim results and make the final decision to terminate the trial	14+16
Harms	22	Plans for collecting, assessing, reporting, and managing solicited and spontaneously reported adverse events and other unintended effects of trial interventions or trial conduct	16
Auditing	23	Frequency and procedures for auditing trial conduct, if any, and whether the process will be independent from investigators and the sponsor	16
**Ethics and dissemination**			
Research ethics approval	24	Plans for seeking research ethics committee/institutional review board (REC/IRB) approval	16
Protocol amendments	25	Plans for communicating important protocol modifications (eg, changes to eligibility criteria, outcomes, analyses) to relevant parties (eg, investigators, REC/IRBs, trial participants, trial registries, journals, regulators)	16
Consent or assent	26a	Who will obtain informed consent or assent from potential trial participants or authorised surrogates, and how (see Item 32)	16
	26b	Additional consent provisions for collection and use of participant data and biological specimens in ancillary studies, if applicable	N/A
Confidentiality	27	How personal information about potential and enrolled participants will be collected, shared, and maintained in order to protect confidentiality before, during, and after the trial	16
Declaration of interests	28	Financial and other competing interests for principal investigators for the overall trial and each study site	20
Access to data	29	Statement of who will have access to the final trial dataset, and disclosure of contractual agreements that limit such access for investigators	20
Ancillary and post-trial care	30	Provisions, if any, for ancillary and post-trial care, and for compensation to those who suffer harm from trial participation	Not provided

For peer review only

Dissemination policy	31a	Plans for investigators and sponsor to communicate trial results to participants, healthcare professionals, the public, and other relevant groups (eg, via publication, reporting in results databases, or other data sharing arrangements), including any publication restrictions	20
	31b	Authorship eligibility guidelines and any intended use of professional writers	20
	31c	Plans, if any, for granting public access to the full protocol, participant-level dataset, and statistical code	20
**Appendices**			
Informed consent materials	32	Model consent form and other related documentation given to participants and authorised surrogates	Supplementary material
Biological specimens	33	Plans for collection, laboratory evaluation, and storage of biological specimens for genetic or molecular analysis in the current trial and for future use in ancillary studies, if applicable	N/A

*It is strongly recommended that this checklist be read in conjunction with the SPIRIT 2013 Explanation & Elaboration for important clarification on the items. Amendments to the protocol should be tracked and dated. The SPIRIT checklist is copyrighted by the SPIRIT Group under the Creative Commons "Attribution-NonCommercial-NoDerivs 3.0 Unported" license.

For peer review only
